# Analysis of epidemiological trends in chronic diseases of Chinese residents

**DOI:** 10.1002/agm2.12134

**Published:** 2020-11-29

**Authors:** Hu Shilian, Wang Jing, Cheng Cui, Wu Xinchun

**Affiliations:** ^1^ Gerontology Institute of Anhui Province The First Affiliated Hospital of USTC, Division of Life Sciences and Medicine. University of Science and Technology of China Hefei 230001 China; ^2^ Anhui Provincial Key Laboratory of Tumor Immunotherapy and Nutrition Therapy Hefei China

**Keywords:** China, chronic diseases, epidemiological research, health conditions, spectrum of disease

## Abstract

Following the population aging in China, dramatic changes have been observed in the spectrum of diseases among Chinese residents. E.g., the incidence and prevalence of chronic diseases, such as cardiovascular, cerebrovascular, metabolic, and respiratory diseases, are constantly growing. Additionally, osteoporosis, sarcopenia, and chronic renal disease have gradually become common chronic diseases among the elderly. Chronic diseases in the elderly have ranked first in the spectrum of diseases among Chinese residents. Therefore, understanding the trends of main chronic diseases among Chinese residents and developing proactive countermeasures have become a major public health issue for China.

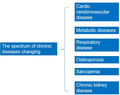

## 1 INTRODUCTION

In August 2016, Chinese General Secretary Xi Jinping made important arrangements for a “Healthy China” at the National Health Conference convened in Beijing, and proposed making health a strategic priority to accelerate public health and fitness in China.^1^, ^2^ In October 2016, the Central Committee of the Communist Party of China and the State Council promulgated the Outline of Healthy China 2030 Plan, which specified that the core concept in prioritizing health is a more accurate positioning for the development of health and fitness in the long run as it focuses on the all‐round development and health of a greater number of people.^1^ People’s health management has become an important national strategy because of the great importance attached to health by China’s policy‐makers.^3^ Due to social and economic development, progress in medical and health services, and changes in people’s lifestyles, the spectrum of disease among Chinese people has profoundly changed. A growing number of Chinese residents are contracting chronic diseases that are closely associated with environmental factors and unhealthy lifestyles. Five chronic diseases—cardiovascular diseases, tumors, diabetes, respiratory diseases, and mental disorders—have been identified as having a far‐reaching impact on the country’s medical and economic systems over the next two decades, according to a renewed warning by the Global Risks Report 2011.^2^ The deaths caused by chronic diseases in China account for 87% of total deaths. The burden of chronic diseases has accounted for approximately 70% of the total burden of diseases in China, thus posing a tough challenge for the prevention and treatment of chronic diseases.^4^ Additionally, nearly 300 million Chinese people suffer from hypertension and being overweight. It has been estimated that approximately 100 million Chinese people contract diabetes and chronic obstructive pulmonary disease (COPD) and more than 10 million people suffer from stroke, coronary heart disease (CHD), and obesity, respectively. These diseases not only consume considerable medical resources, but also place a heavy burden on individuals, their families, and society.^2^ Therefore, obtaining a clear perspective on the epidemiological trend of chronic diseases among Chinese residents will provide a foundation for exploring strategies for the prevention and treatment of such diseases in China and for proactively preventing and controlling the incidence and development of such diseases.

## 2 CHRONIC CARDIOVASCULAR DISEASE

The prevalence of cardiovascular diseases in China is on the rise. According to the survey of the *Cardiovascular Disease Report in China 2018* released by China’s National Center for Cardiovascular Disease,^5^ approximately 290 million people contracted cardiovascular diseases in China, including 250 million people with hypertension and 11 million people with CHD. Meanwhile, the medical expenses related to cardiovascular diseases have rapidly grown, with the growth rate exceeding that of GDP in recent years.^6^

According to the 2013 Fifth National Health Services Survey of China,^7^ around 1.2% of people aged over 15 years and about 2.8% of people aged over 60 years contracted CHD. Based on the data of the People’s Republic of China’s Sixth National Population Census,^8^ it was estimated that more than 11 million people aged over 15 years suffered from CHD in 2013. From 1999 to 2013, 81/100 000 people per year contracted acute myocardial infarction in Tianjin, and the incidence was declining year by year, according to a 15‐year epidemiological study on acute myocardial infarction in Tianjin.^9^ Nevertheless, the same study reported that the incidence of acute myocardial infarction in people aged younger than 45 years was rising year by year, especially among the rural population. In 2002, the mortality rate of CHD was 40/100 000 urban residents per year and 28/100 000 rural residents per year in China, which increased to 101/100 000 per year and 99/100 000 per year, respectively, in 2001. These data prove that the mortality rate for CHD in China was surging, with that of the rural population approaching that of the urban population.^10^ According to data from the *China Health and Family Planning Statistical Yearbook 2017*, the mortality rate for CHD among the rural and urban populations in 2016 maintained the growing trend that was initially observed for 2013. Specifically, the death rate for CHD was 113/100 000 urban residents per year and 118/100 000 rural residents per year in 2016; thus, the death rate for CHD among the rural population has surpassed that among the urban population.

The significance of controlling blood pressure was highlighted in both the ^11^ and the “Outline of Healthy China 2030 Plan,”^12^ which identified management of hypertension as a priority for prevention and treatment of chronic disease in China. Evidence‐based medicine studies suggest that hypertension has become one of the most significant risk factors for cardiovascular and cerebrovascular diseases among the Chinese, and is associated with more than half of the incidence and mortality related to cardiovascular and cerebrovascular diseases.^13^ Furthermore, more Chinese residents died of cardiovascular and cerebrovascular diseases than other reasons during these years,^14^ so such diseases are regarded as major threats to the health of Chinese residents. As a result, curbing hypertension is the crux in the prevention and treatment of cardiovascular and cerebrovascular diseases. The crude prevalence (adjusted) rates of the population aged 15 years and older in five national sample surveys on hypertension conducted in China in 1958‐1959, 1979‐1980, 1991, 2002, and 2012 were 5.1%, 7.7%, 13.6%, 18.8%, and 25.2%, respectively. It can be concluded that the prevalence of hypertension among Chinese adults has clearly been on the rise, despite the discrepancy in the survey scope, total respondents, age, and diagnostic criteria.^15^ Additionally, according to the data from surveys on hypertension collected from 450 000 people across 31 provinces, municipalities, and autonomous regions in China from 2012 to 2015, the crude prevalence rate of hypertension among the population aged 18 years and older was 27.9%. Furthermore, the data also showed that the prevalence of hypertension in large‐ and medium‐sized cities remained high. At the same time, it substantially increased among the rural population, where it reached even higher levels than those observed among the urban population. The prevalence of hypertension among adults substantially increases as they age. For example, nearly one‐third of the population aged 45‐59 years, and half of the elderly suffer from hypertension. Nevertheless, it is worrisome that the rates of awareness, treatment, and control of hypertension are very low while the prevalence, disability, and mortality rates are high. Such rates of hypertension in Chinese adults sharply increased as the policy on medical reform was put in place during recent years. For example, China’s hypertension survey from 2012 to 2015^16^ showed that the rates of awareness, treatment, and control of hypertension among residents aged 18 years and older were 46.9%, 40.7%, and 15.3%, respectively. Despite an increase in such rates in 2002, the rates of awareness and treatment of hypertension were <50%, and the rate of control was even lower, far lagging behind those in developed countries.^17^

The prevalence of hypertension in China continues to increase and is not effectively controlled. Furthermore, hypertension is an important risk factor for cardiovascular and cerebrovascular diseases. It is anticipated that the prevalence of hypertension and its cardiovascular and cerebrovascular complications in China will continue to rise over an extended period of time. As a result, it is all the more urgent to increase efforts in the prevention and treatment of hypertension, which is expected to be one of the most crucial public health issues among Chinese residents in coming years. As mentioned, the mortality rate for CHD in rural residents has substantially increased, surpassing that of urban residents. Furthermore, China has an expansive agricultural industry with a large rural population and high morbidity, so it should make every endeavor to enhance the prevention and treatment of CHD for rural residents.

## 3 CEREBROVASCULAR DISEASES

Stroke is the second‐leading cause of death among those 60 years and older in China, and has become a global public health issue due to its high rates of incidence, disability, and mortality.^18^ Over recent years, China has witnessed a rapid development of cerebrovascular diseases, with high prevalence and disability rates and increased medical expenses. Consequently, such diseases have placed an enormous financial burden on individuals, families, and society. China conducted a large‐scale epidemiological survey on cerebrovascular diseases as early as in the 1980s, which was comparable to the epidemiological surveys conducted over recent years due to the basic consistency in survey methods and diagnostic criteria. In 1982, the age‐standardized prevalence of stroke among residents in six major Chinese cities was 719/100 000, the incidence was 205/100 000 people per year, and the mortality rate was 109/100 000 people per year. In 1985, the age‐standardized prevalence of stroke among rural residents in 21 Chinese provinces and cities was 394/100 000, the incidence was 172/100 000 people per year, and the mortality rate was 132/100 000 people per year.^19^, ^20^

The prevalence (crude) rate of stroke among people aged over 60 years in 1992 was 7.9%, which increased to 12.7% in 2000, according to a cross‐sectional survey of urban and rural residents in Beijing in 1992 and 2000.^21^ During the “Twelfth Five‐Year Plan,” the Beijing Neurosurgical Institute teamed up with the Chinese Center for Disease Control and Prevention in performing a large‐scale cross‐sectional survey of cerebrovascular diseases among 600 000 people in 2003.^22^ The survey indicated that the prevalence (crude) rate of stroke in China was around 1287/100 000, which was a dramatic growth from 30 years ago. Furthermore, the prevalence of stroke among men over 40 years was significantly higher than that among women over 40 years, and that among the rural population gradually overtook that among the urban population. Currently, approximately 11 million patients suffer from stroke in China, aside from asymptomatic cerebral infarction, according to the estimate of prevalence by this epidemiological survey. In light of an epidemiological survey on cerebrovascular diseases among 600 000 Chinese residents conducted in 2013, the incidence (crude) rate of stroke was 274/100 000 people per year. Specifically, the incidence (crude) rates of stroke among the rural and urban populations were 227/100 000 people per year and 168/100 000 people per year, respectively, while the prevalence of asymptomatic cerebral infarction was about 19.9%. Compared with three decades ago, the incidence of stroke among rural residents sharply increased by 32%, whereas among urban residents, it had increased by 18.1%. Furthermore, people were suffering from stroke at an earlier age.^20^, ^22^ In the meantime, this survey demonstrated that the death (crude) rate of stroke was 126/100 000 people per year. Specifically, the death (crude) rates among rural and urban populations were 117/100 000 people per year and 75/100 000 people per year, respectively, which was 11% and 31% lower compared to 30 years ago. Additionally, an epidemiological study among 15 000 urban residents in Tianjin that lasted for over two decades^23^ illustrated that the average annual incidence rate of an individual’s first stroke in 1992‐1998, 1999‐2005, and 2006‐2012 was 124/100 000 people per year, 190/100 000 people per year, and 318/100 000 people per year, respectively. It was estimated that the incidence would grow by about 6.5% a year, with the greatest increase in men aged 45‐65 years.

An epidemiological survey of cerebrovascular diseases among 600 000 Chinese people in 2013^20^, ^22^ showed that ischemic stroke, cerebral hemorrhage, and subarachnoid hemorrhage represented 70%, 24%, and 4% of the total strokes, respectively. In addition, the incidence of a cerebral hemorrhage in China was far higher than that in the United States. The incidence rates of ischemic stroke among men and women were 182/100 000 people per year and 152/100 000 people per year, respectively; those of cerebral hemorrhage among men and women were 70/100 000 people per year and 63/100 000 people per year, respectively; those of subarachnoid hemorrhage among men and women were 12/100 000 people per year and 8/100 000 people per year, respectively; and those of transient ischemic attack (TIA) among men and women were 103/100 000 people per year and 24/100 000 people per year, respectively, with estimated TIA patients being 1.35 million and new TIA patients being 310 000 in China. According to the 2010 China Chronic Disease and Risk Factor Surveillance (CCDRFS), the age‐standardized prevalence of TIA in China was around 2%, while the awareness and treatment rates were <10%. Additionally, TIA was a precursor to stroke, so proactive intervention could effectively prevent the incidence of stroke.^24^ By 2013, the age‐standardized mortality of stroke among Chinese residents had dropped by 21% from 1990. Specifically, the mortality of ischemic stroke increased by 29%, while that of hemorrhagic stroke dropped by 38%, which may be due to the prevention and treatment as well as primary prevention of hypertension.

The prevalence of cerebrovascular diseases in China remains high and continues to rise; however, the mortality rate seems to have dropped, due to China’s proactive prevention of cerebrovascular diseases. It is noteworthy that the age at onset of stroke has reduced in China, indicating that attention should be paid to the elderly and also to the prevention and treatment of cardiovascular and cerebrovascular diseases among young and middle‐aged residents. More proactive and effective strategies should be developed for screening, awareness, prevention, and treatment of TIA. Given the rapid increase in the incidence of cerebrovascular diseases among rural residents, great importance should also be attached to the intervention mode among rural populations to minimize the damage of such diseases to the health of Chinese residents as well as the society and economy.

## 4 ENDOCRINE AND METABOLIC DISEASES

Over the past four decades, due to the aging of the Chinese population and changes in lifestyles, diabetes has developed from a rare disease into a common and frequently occurring disease. China has carried out plenty of large‐scale epidemiological surveys on diabetes nationwide and the prevalence of diabetes soared to 10.4% in 2013 from 0.67% in 1980. In 1980, the epidemiological data from 300 000 people across 14 Chinese provinces and cities suggested that the prevalence of diabetes among all age groups was only 0.7%. From 1994 to 1995, the epidemiological surveys on diabetes among 210 000 people in 19 Chinese provinces and cities indicated that the prevalence of diabetes among people aged 25‐64 years was 2.5%. In 2002, the national nutrition survey in China showed that the prevalence rates of diabetes among urban and rural residents over 18 years old were 4.5% and 1.8%, respectively. From 2007 and 2008, the Chinese Diabetes Society performed epidemiological surveys on diabetes across 14 counties across China, estimating that 9.7% of adults over 20 years old in China suffered from diabetes.^25^ In 2013, the CCDRFS conducted by the Chinese Center for Disease Control and Prevention suggested that 10.4% of Chinese people aged 18 years and over developed diabetes and that the prevalence of prediabetes was up to 35.7%.^26^ About 140 million people had diabetes, according to the People’s Republic of China’s Sixth National Population Census.^8^ China had become the leading country with the most diabetic patients in the world. Worse still, 63% of them were unable to receive effective early treatment and education due to lacking a diagnosis.^25^ Furthermore, the prevalence of diabetes may continue to rise as population aging accelerates in China. Epidemiological surveys in 2008 and 2013 suggested that more than 20% of the elderly over 60 years in China are likely to develop diabetes.^25^ On the whole, the prevalence of diabetes and prediabetes is on the rise in China, so the prevention and control of diabetes represent tough challenges.

Over the past 20 years, the prevalence of overweight and obesity has surged in China, seriously harming the health of residents. According to data from the China Health and Nutrition Survey (CHNS), the proportion of overweight and obese adults in China rose from 25.1% in 1997 to 39.6% in 2009. Furthermore, the prevalence of abdominal obesity among adults grew from 18.6% to 37.4%, and the proportion of adults with overweight, obesity, and abdominal obesity surged.^27^ The overweight and obesity rates of Chinese residents have dramatically increased over the past two decades. According to the *Report on Chinese Residents’ Chronic Diseases and Nutrition (2015)*,^28^ the monitoring results of Chinese residents’ nutrition and health showed that the overweight and obese residents aged 18 years and older accounted for up 42.0% of the total, which was close to the level of many developed countries, such as the United States and the United Kingdom. Specifically, the overweight and obesity rates were 30.1% and 11.9%, respectively, which were 7.3% and 4.8% higher compared to 2002. The increase in overweight and obesity rates of the rural population was significantly higher compared to the urban population, implying that emphasis should be given to the overweight and obesity of rural residents. Overweight and obesity contribute to the risk of diverse chronic diseases, such as cardiovascular and cerebrovascular diseases, endocrine and metabolic diseases, and a range of malignant tumors. In 2011, 30.4% of men aged 18‐65 years and 28.1% of women in the same age group had central obesity,^27^ suggesting the acute challenges posed by overweight and obesity in China.

In line with the 2002 CHNS,^29^ the prevalence of dyslipidemia among Chinese residents older than 18 years was 18.6%, whereas the prevalence rates of hypercholesterolemia and hypertriglyceridemia were 2.9% and 11.9%, respectively. According to the 2011 CHNS,^30^ the prevalence of dyslipidemia among Chinese adults was 39.9%, whereas the prevalence rates of hypercholesterolemia and hypertriglyceridemia were 9.0% and 27.0%, respectively. It can be observed that the prevalence of dyslipidemia in Chinese adults has significantly risen over the last decade. The 2013‐2014 CCDRFS investigated 160 000 residents across 31 provinces, cities, and autonomous regions across China.^31^ The results showed that the total serum cholesterol (TC) and low‐density lipoprotein cholesterol (LDL‐C) of residents aged 18 years or over were 4.70 mmol/L and 2.88 mmol/L, respectively. Furthermore, the TC level had significantly increased as compared with the 2002 CHNS^29^ (3.81 mmol/L). An epidemiological survey on 43 000 residents across 13 Chinese provinces and cities^32^ demonstrated that the rates of awareness, treatment, and control among residents over 18 years old were 31.0%, 19.5%, and 8.9%, respectively. Blood lipids were closely associated with the prevention and treatment of cardiovascular and cerebrovascular diseases, and effectively reducing the levels of TC and LDL‐C has become a core strategy for the primary and secondary prevention of cardiovascular diseases in China.

Over the past decades, the prevalence of metabolic‐related chronic diseases (eg, diabetes, obesity, and hyperlipidemia) has remained high and shows a clear onward trend. It is therefore anticipated that such diseases will remain common chronic diseases among Chinese residents in future. Not only does this represent a grave threat to health, it also increases medical expenses and the burden on medical care, society, and the economy. Accordingly, the current focus should be on the control of metabolic‐related chronic diseases and on minimizing the incidence of relevant complications. Lowering the incidence and prevalence of such diseases remains the primary goal in upcoming years.

## 5 RESPIRATORY DISEASES

Chronic respiratory diseases, represented by chronic obstructive pulmonary disease (COPD), result in a heavy economic burden. It was estimated that COPD would become the third leading global cause of death by 2020, second only to ischemic heart disease and cerebrovascular disease. As the population is aging faster than ever, COPD has also become one of the principal causes of disability and death among senior citizens in China, bringing considerable economic and social burdens. The Global Burden of Disease indicated that the disability‐adjusted life years lost to COPD was estimated to surpass 2000/100 000 people in China,^33^ thereby adversely affecting the prognosis and quality of life in middle‐aged and elderly patients. Chan et al^34^ employed logistic regression and collected and analyzed the data on the prevalence of COPD among Chinese residents from 1990 to 2010 from relevant epidemiological studies on the population with COPD in China. The results were as follows: the prevalence rates of COPD in the populations aged under 20 years and over 80 years were 0.5% and 21.0%, respectively, in 1990, which increased to 0.6% and 22.9%, respectively, in 2010. In addition, such prevalence rose with age, and the prevalence of male residents was significantly higher than that of female residents. The past two decades saw an increase of about two‐thirds in patients with COPD in China. Tang et al^35^ analyzed a total of 118 000 subjects from 49 reports on the epidemiological surveys on COPD in China from 2000 to 2014. They concluded that the prevalence of COPD in Chinese residents over 40 years old was 9.3%, and that prevalence in men was nearly twice that of women. Furthermore, the incidence of COPD among Chinese residents over 40 years old was 8.2%, according to an epidemiological survey on COPD in seven Chinese provinces and cities between 2002 and 2004.^36^ According to the “Chinese Expert Consensus on Anti‐Infective Therapy for Acute Exacerbation of Chronic Obstructive Pulmonary Disease,” the prevalence rates of COPD among Chinese residents over 40 years old and over 60 years old were 13.7% and over 27.0%, respectively. The total patients with COPD were estimated to be 100 million. Hence, like hypertension and diabetes, COPD has become a common chronic disease.^37^, ^38^ Multiple epidemiological surveys across different regions between 2002 and 2015 indicated that the diagnosis rate of COPD was 23.6%‐30.0% in China and that only half of the patients with COPD received treatment.^39^ The 2015 China Pulmonary Health Study^38^ selected and investigated some 57 000 residents from 10 provinces/autonomous regions in China, reporting that only 2.6% of patients with COPD were aware of their disease. Li et al^40^ analyzed a third of China’s retrospective samplings and surveys on causes of death. They found that (non‐infectious) respiratory diseases accounted for 15.8% of the total deaths, thus representing the third leading cause of death, where the main respiratory disease was COPD. Currently, frontline doctors have less awareness of the prevention and treatment of chronic respiratory diseases, particularly COPD, compared to other chronic diseases, such as hypertension and diabetes. On top of that, the basic health services for patients with COPD should be improved in China, as should the prevention and management of COPD.^39^ The prevention and treatment of chronic respiratory diseases constitute an integral part of the “Healthy China Action Plan” (2019‐2030), and every citizen should have a role in this plan.^39^

With an ever‐aging population in China, the prevalence of chronic respiratory diseases dominated by COPD has been rapidly increasing. Consequently, COPD has become one of the principal causes of death among Chinese residents. Nonetheless, the awareness of prevention and treatment of COPD is rather weak among Chinese residents. Accordingly, China needs to enhance general knowledge on chronic respiratory diseases like COPD, reinforce disease control, improve the quality of life of patients with COPD, and take precautions against chronic respiratory diseases (mainly COPD).

## 6 MUSCULOSKELETAL DISEASES

The prevalence of osteoporosis varies among different regions and ethnic groups in China, and such disease may lead to severe complications, such as falls and fractures. The results of a sampling survey conducted across China from 1999 to 2000 among Han residents were as follows: the prevalence rates of osteoporosis based on the bone mineral density (BMD) values of the vertebral body and femoral neck were 9.9% and 11.1%, respectively, in those aged over 40 years; the prevalence rates among those aged over 60 years were 14.2% and 13.2%, respectively.^41^ The results of an epidemiological survey on osteoporosis across China from 2003 to 2006 were as follows: the bone mass of men peaked between 20 and 30 years old, while that of women peaked between 30 and 40 years old. Furthermore, the total prevalence of osteoporosis among Han residents over 40 years old was 15.2%, and the prevalence rates of osteoporosis based on the BMD values of the vertebral body and femoral neck were 19.7% and 14.1%, respectively. The prevalence among women was significantly higher than among men.^42^ In 2018, the Chinese Center for Disease Control and Prevention collaborated with the Chinese Society of Osteoporosis and Bone Mineral Research. It carried out an epidemiological survey on osteoporosis among 20 000‐odd residents in 11 Chinese provinces and cities. The survey indicated that the prevalence rates of osteopenia and osteoporosis among Chinese residents aged 40‐49 years were 32.9% and 3.2%, respectively; those of osteopenia and osteoporosis among Chinese residents aged over 50 years were 46.4% and 19.2%, respectively; and the prevalence of osteoporosis among Chinese residents aged over 65 years was 32.0%, where women and rural residents had significantly higher rates than men and urban residents.^43^ The prevalence of osteoporosis in Chinese residents was high, while their awareness of such disease was seriously inadequate. The survey demonstrated that the awareness rates of osteoporosis among patients aged 40‐49 years and over 50 years were 0.9% and 7.0%, respectively. The detection rates of BMD and awareness rates of osteoporosis were low because residents, especially the senior residents, had an insufficient understanding of the importance and harm of osteoporosis. Moreover, they received treatment only when enduring serious complications, thereby missing the best opportunity for the prevention and treatment of osteoporosis.

The mass and strength of skeletal muscles vary with age, peak in young and middle ages, and then drop as one gets older. Specifically, muscle mass decreases by 1%‐2% after 50 years of age, while muscle strength declines by 1.5%‐3%. Furthermore, the total amount of muscle is reduced by 30% by 80 years and is accompanied by greater reductions in muscle power and strength.^44^ The prevalence of sarcopenia among the elderly in Asia is 8%‐35%. Currently, there is no nationwide epidemiological information on sarcopenia in China; there are differences in the epidemiological data on sarcopenia in some regions in China because of the differences in measurement methods and subjects. Cheng et al^45^ adopted dual‐energy X‐ray absorptiometry to measure BMD of 3544 Shanghai residents aged 18‐96 years, indicating that the prevalence rates of sarcopenia in men and women over 70 years were 13.2% and 4.8%, respectively. Furthermore, the survey by Zhang et al^46^ suggested that the overall prevalence of sarcopenia among 1227 Shanghai residents was 14.3%, whereas the prevalence rates of sarcopenia among men and women were 14.9% and 14.0%, respectively. In their survey, Yang et al^47^ illustrated that the prevalence of sarcopenia among senior citizens in Jiangsu Province was 28.8%. Additionally, the survey conducted in Tianjin by Han et al^48^ encompassing 1069 senior residents who were over 60 years old suggested that the prevalence rates of sarcopenia among male and female residents were 6.4% and 11.5%, respectively. These rates were 9.3% and 4.1% among elderly men and women in Taiwan, China, respectively.^49^ Wu et al^50^ performed a meta‐analysis on 18 570 subjects from 2010 to 2018, which demonstrated that the prevalence of sarcopenia among elderly Chinese residents was 12%. Plus, the subgroup analysis suggested that the prevalence of sarcopenia among elderly residents in mainland China was 17%, whereas that of sarcopenia among elderly residents in Hong Kong and Taiwan was 6%. In response to the serious situation of sarcopenia in the elderly, it is necessary to obtain a clear idea of the severity of sarcopenia‐related effects on chronic diseases and quality of life among senior citizens, and to prevent and reverse the development of sarcopenia, considering the large population base and accelerated aging of the population in China. In addition, efforts should be made to maintain muscle mass, strength, and function in the elderly, and enhance their quality of life.^44^

Elderly citizens in China have experienced a rapid increase in the incidence and prevalence of chronic musculoskeletal diseases, such as osteoporosis and sarcopenia. In view of the ever‐worsening aging of the population in China, the rates of elderly Chinese patients with osteoporosis and sarcopenia are expected to rise, eventually becoming the main chronic diseases in China over the upcoming years. Proactive actions against osteoporosis and sarcopenia should be made, for these two diseases may cause severe complications, such as falls, fractures, and asthenia, adversely impacting the quality of life, and imposing an onerous burden on an aging society.

## 7 DISEASES OF THE KIDNEY SYSTEM

According to an epidemiological survey of 47 000 people across 13 Chinese provinces and cities in 2009,^51^ the standardized and unstandardized prevalence rates of chronic kidney disease (CKD) were 10.8% and 12.8%, respectively. In 2018, Wang et al^52^ performed a meta‐analysis of 22 papers on the prevalence of CKD in 240 000 healthy people from 2007 to 2017. They found that the unstandardized prevalence of CKD was 12.5%, indicating a high prevalence of CKD in China. The prevalence rates of CKD in elderly residents aged 60 to 69 years, 70 to 79 years, and 80 years in Beijing were 20.8%, 30.5%, and 37.8%, respectively,^53^ which implied that the prevalence of CKD gradually increased with age. According to the “National Dialysis Transplantation Registration Report in 1999,”^54^ among the Chinese patients with uremia receiving dialysis, those with glomerulonephritis and diabetic nephropathy accounted for 49.9% and 13.3%, respectively. However, the proportion of patients with diabetic nephropathy has increased as the prevalence of diabetes has increased rapidly in China over the past three decades. In 2016, Zhang et al^55^ analyzed the trend of the CKD spectrum in China from 2010 to 2015 using data from the general population and hospitalized population in China. They first analyzed the 35.3 million patients hospitalized in Grade III hospitals in China between 2010 and 2015, showing that the proportion of diabetic nephropathy was lower than that of glomerulonephritis among hospitalized patients in 2010. However, this situation gradually changed, with the former starting to exceed the latter from 2011, followed by an increasing difference over the following years. In 2015, the proportions of diabetic nephropathy and glomerulonephritis cases were 1.1% and 0.7%, respectively. Furthermore, analysis of the data obtained from adult residents over 18 years revealed that the percentage of diabetic nephropathy was higher than that of glomerulonephritis. Specifically, the percentages of diabetic nephropathy and glomerulonephritis were 1.6% and 0.7%, respectively, in 2015. Approximately 21.3% of the general population contracted both diabetes and diabetic nephropathy. Based on the 140 million patients with diabetes in China, it has been estimated that there are 30 million patients with diabetic nephropathy in China. Due to the growing number of patients with diabetes, diabetes‐related CKD has overtaken glomerulonephritis‐related CKD as the leading cause of CKD in China. End‐stage renal disease (ESRD) results from the irreversible decline in renal function when different kidney diseases reach an advanced state. As a result, water, electrolytes, and metabolic waste build up in the body, so renal replacement therapy (RRT) is required. China’s surveys on ESRD have focused on patients receiving RRT, which commenced in 1999 when there were 42 000 patients with ESRD in China, with a prevalence of 33.6/1 000 000 and an incidence of 15.3/1 000 000. According to the second national survey by the Chinese Medical Doctor Association in 2008, the patients with ESRD reached 65 000 in China by 2007, resulting in a prevalence of 51.7/1 000 000.^56^ In 2008, the number of patients with ESRD grew to 103 000 in 2008, and the prevalence was 79.1/1 000 000.^56^ Thereafter, the incidence of ESRD surged in China, and the patients receiving RRT exceeded 300 000 by the end of 2014.^57^

Over recent years, the prevalence of CKD has been on the rise in China. Especially high rates were observed among senior citizens due to their advanced age, thereby substantially affecting their health and quality of life. In addition to enhancing awareness and curbing the diseases of the kidney system, China should take proactive measures to control diseases that are likely to cause CKD complications, such as hypertension and diabetes, administer drugs rationally, and reduce the damage to the kidney system.

## 8 SUMMARY

Health is a crucial aspect of human development that provides a basis for economic and social development. Improvement in health care represents an important achievement in our efforts to build a stronger nation and achieve greater prosperity. It is also a shared expectation of the public.^2^ As China becomes an aging society due to the rapidly aging population, the lifelong health of all is the ultimate goal. Recent years have witnessed numerous changes in the spectrum of disease among Chinese residents. The incidence and prevalence of chronic diseases, such as cardiovascular, cerebrovascular, metabolic, and respiratory diseases, are constantly growing. Additionally, osteoporosis, sarcopenia, and chronic renal disease have gradually become common chronic diseases among the Chinese elderly. Chronic diseases in the elderly have ranked first on the spectrum of disease for Chinese residents. Therefore, understanding the trends in main chronic diseases among Chinese residents, developing proactive countermeasures, and providing health services for all, particularly for the elderly, are national strategies that are currently being implemented. The *Medium and Long‐term Plan on Prevention and Control of Chronic Diseases (2017‐2025)* has established the principles for the prevention and treatment of chronic diseases in China. When the strategy of “Healthy China” is put in place, it is necessary to grasp the epidemiological trends of chronic diseases and the status of prevention and treatment in China and to explore solutions and countermeasures in a bid to offer references for the prevention and treatment of such diseases.^4^

## 9 ACKNOWLEDGMENTS

This study was funded by the Anhui Provincial Project of the Key Laboratory of Tumor Immunotherapy and Nutrition Therapy (2019b12030026).

## CONFLICTS OF INTEREST

Nothing to disclose.

## AUTHOR CONTRIBUTIONS

Hu Shilian supervised the study and obtained the financial support. Wang Jing conducted the literature search. Cheng Cui directed the article writing. Wu Xinchun prepared and revised the manuscript.
